# The DELLA proteins interact with MYB21 and MYB24 to regulate filament elongation in *Arabidopsis*

**DOI:** 10.1186/s12870-020-2274-0

**Published:** 2020-02-07

**Authors:** Huang Huang, Yilong Gong, Bei Liu, Dewei Wu, Min Zhang, Daoxin Xie, Susheng Song

**Affiliations:** 1 Beijing Advanced Innovation Center for Tree Breeding by Molecular Design, Beijing University of Agriculture, Beijing, 102206 China; 2 Beijing Key Laboratory of Plant Gene Resources and Biotechnology for Carbon Reduction and Environmental Improvement, RNA Center, College of Life Sciences, Capital Normal University, Beijing, 100048 China; 3 Jiangsu Provincial Key Laboratory of Crop Genetics and Physiology, Yangzhou University, Yangzhou, 225009 China; 4 School of Life Sciences, Tsinghua University, Beijing, 100084 China

**Keywords:** Gibberellin, Jasmonate, MYB21, MYB24, Filament elongation

## Abstract

**Background:**

Gibberellin (GA) and jasmonate (JA) are two essential phytohormones for filament elongation in *Arabidopsis*. GA and JA trigger degradation of DELLAs and JASMONATE ZIM-domain (JAZ) proteins through SCF^SLY1^ and SCF^COI1^ separately to activate filament elongation. In JA pathway, JAZs interact with MYB21 and MYB24 to control filament elongation. However, little is known how DELLAs regulate filament elongation.

**Results:**

Here we showed that DELLAs interact with MYB21 and MYB24, and that R2R3 domains of MYB21 and MYB24 are responsible for interaction with DELLAs. Furthermore, we demonstrated that DELLA and JAZ proteins coordinately repress the transcriptional function of MYB21 and MYB24 to inhibit filament elongation.

**Conclusion:**

We discovered that DELLAs interact with MYB21 and MYB24, and that DELLAs and JAZs attenuate the transcriptional function of MYB21 and MYB24 to control filament elongation. This study reveals a novel cross-talk mechanism of GA and JA in the regulation of filament elongation in *Arabidopsis*.

## Background

Stamen, comprising a filament and an anther, is one of the plant reproductive organs. Disruptions in stamen development, such as in filament elongation, anther dehiscence, or pollen maturation, can cause male sterility [1, 2]. Numerous studies revealed that these processes are controlled by phytohormones, including jasmonate (JA) and gibberellin (GA) [3, 4].

JAs, including jasmonic acid and its oxylipin derivatives, play key roles in the regulation of plant developmental processes, such as root growth [5], stamen development [6], trichome initiation [7, 8], flowering [9], leaf senescence [10, 11], and apical hook formation [12–14], and as well as control diverse defense responses to abiotic and biotic stresses [15–21]. JAs are perceived by F-box protein CORONATINE INSENSITIVE 1 (COI1) [22, 23],and subsequently induce the degradation of JASMONATE ZIM-DOMAIN (JAZ) proteins [24–26]. Mutants in *Arabidopsis* JA biosynthesis and signaling transduction, such as *defective in anther dehiscence 1* (*dad1*), *13-lipoxygenase 3/4* (*lox3 lox4*), *12-oxophytodienoate reductase 3* (*opr3*), *coi1–1*, or overexpressing the JAZ dominant forms JAZ1∆3A or JAZ10.4 lead to deficiency in late stamen development, including filament elongation, anther dehiscence and pollen maturation [24, 27–31].

The JA-inducible R2R3 MYB transcription factors MYB21 and MYB24 [32–34], and subgroup IIIe bHLH factors (MYC2, MYC3, MYC4, and MYC5) [5, 35–37] form MYB-MYC complexes to regulate late stamen development [38], and JAZ repressors directly inhibit members of the MYB-MYC complexes [38]. The *myb21 myb24* double mutant displays short filaments, indehiscent anthers, and unviable pollen grains at floral stage 13 [32], and the *myc2 myc3 myc4 myc5* quadruple mutant exhibits delayed filament elongation, anther dehiscence and pollen maturation [38]. JAs act through COI1 to induce degradation of JAZ repressors, which enhances expression of *MYB21* and *MYB24* and releases MYB-MYC complexes to promote late stamen development [22, 24–26, 34, 38].

GAs are cyclic diterpenoid molecules that modulate almost all aspects of plant growth and development, including seed germination [39], stem growth [40], hypocotyl elongation [41, 42], trichome development [43], floral organ development [44], and flowering [45]. In *Arabidopsis*, five DELLA proteins (GIBBERELLIC ACID INSENSITIVE [GAI], REPRESSOR OF GA1–3 [RGA], RGA-like1 [RGL1], RGL2, and RGL3) act as negative regulators by interacting with downstream transcription factors to repress multiple gibberellin responses [46–49]. GAs are perceived by the receptor GIBBERELLIN INSENSITIVE DWARF 1 (GID1a/b/c), which triggers DELLAs ubiquitination by SCF^SLY1^ complex and degradation by the 26S proteasome, and activates downstream transcription factors to control their respective responses [50, 51]. The *Arabidopsis* GA biosynthesis and perception mutants such as *ga1–3*, *gid1a-1 gid1b-1 gid1c-1* are male sterile due to unelongated filament, and delayed anther development [4]. The quadruple mutants Q1 (*ga1–3 gai-t6 rgl1–1 rgl2–1*, wild type for RGA and RGL3) and Q3 (*ga1–3 gai-t6 rgl1–1 rga-t2*, wild type for RGL2 and RGL3) were still retarded in stamen development, while penta mutant (*ga1–3 gai-t6 rga-t2 rgl1–1 rgl2–1*, wild type for RGL3) was fertile, suggesting that DELLA proteins RGA and RGL2 play a principal role in inhibition of stamen development [52].

GAs activate the expression of *MYB21*, *MYB24*, and *MYB57* via suppressing DELLA proteins and up-regulating the expression of JA biosynthetic genes *DAD1* and *LOX1* and JA biosynthesis to mediate filament elongation [52]. Here, in this study we further demonstrated that MYB21 and MYB24 are the direct targets of DELLAs, and act as a necessary node for GA-JA synergism in filament elongation. We showed that DELLAs interact with MYB21 and MYB24 via R2R3 domains, and that DELLA and JAZ proteins coordinately repress the transcriptional function of MYB21 and MYB24 to inhibit filament elongation.

## Results

### MYB21 and MYB24 interact with DELLA proteins

**Fig. 1:**
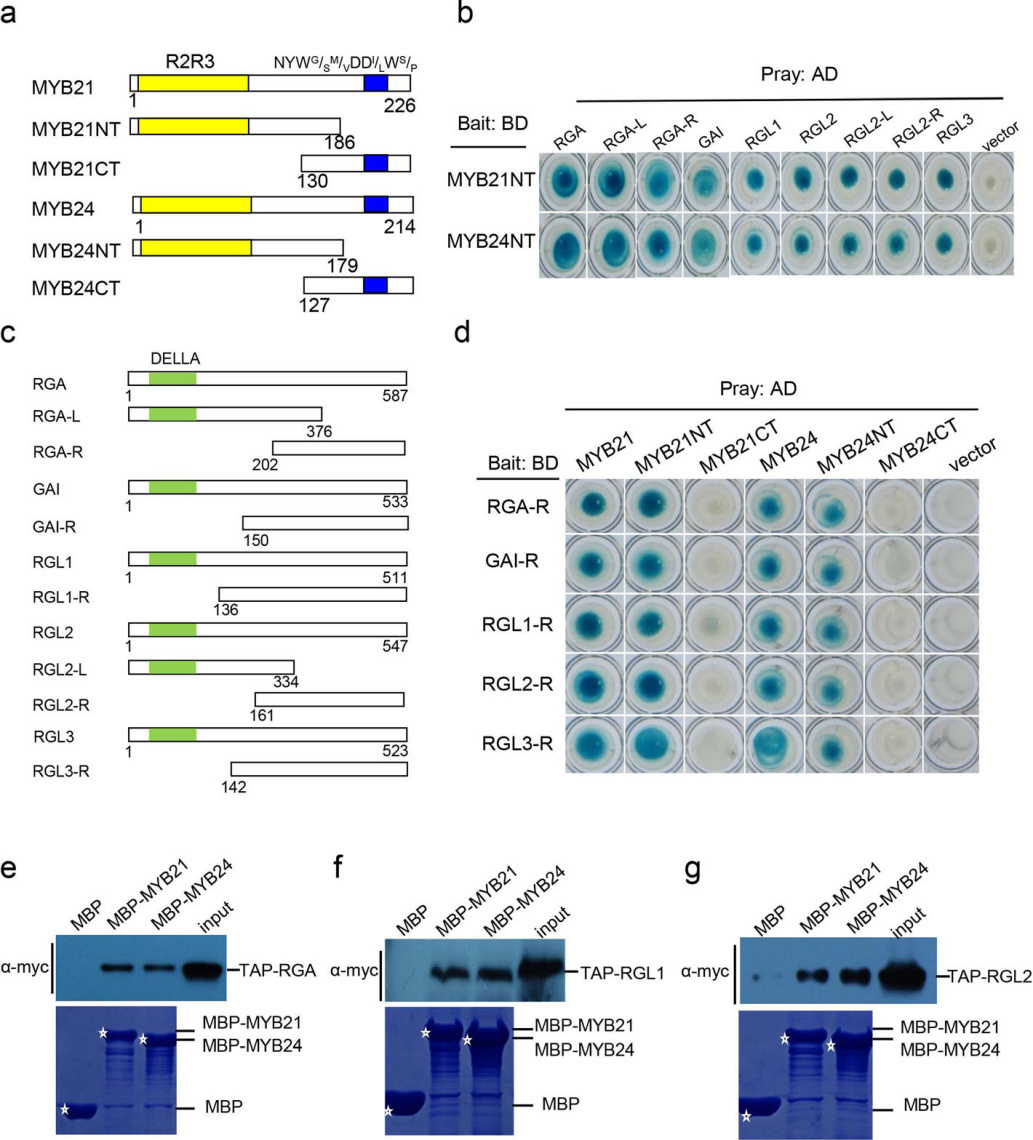
Interactions of DELLAs with MYB21 and MYB24. **a** Schematic diagram of MYB21 and MYB24 domain constructs. The conserved R2R3 domain, and the NYW^G^/_S_^M^/_V_DD^I^/_L_W^S^/_P_ motif are indicated by yellow and blue respectively. The numbers indicate positions of the first and the last amino acid of the domain constructs. **b** Yeast Two-Hybrid (Y2H) assay to detect interactions of MYB21NT and MYB24NT with DELLAs and their derivatives. MYB21NT and MYB24NT were individually fused with the LexA DNA binding domain (BD) in pLexA. DELLAs and their derivatives were individually fused with the activation domain (AD) in pB42AD. Interactions of MYB21NT and MYB24NT with the AD domain in pB42AD empty vector were used as negative controls. Interactions (represented by blue color) were assessed on 2% Gal/1% raffinose/SD/−Ura/−His/−Trp/−Leu/X-β-Gal medium. **c** Schematic diagram of DELLAs domain constructs. The R part of DELLAs contain the conserved DELLA domain (green). The numbers indicate positions of the first and the last amino acid of the domain constructs. **d** Y2H assay to detect interactions of R domains of DELLAs with MYB21, MYB24, and their derivatives. R domains of DELLAs were individually fused with BD domain in pLexA. MYB21, MYB24 and their derivatives were individually fused with the AD domain in pB42AD. Interactions of R domains of DELLAs with the AD domain in pB42AD empty vector were used as negative controls. Interactions were assessed on 2% Gal/1% raffinose/SD/−Ura/−His/−Trp/−Leu/X-β-Gal medium. **e-g** In vitro pull-down assay to detect the interactions of RGA (e), RGL1 (f), RGL2 (**g**) with MYB21 and MYB24. Purified MBP, MBP-MYB21, and MBP-MYB24 fusion proteins were incubated with the TAP-RGA, TAP-RGL1, or TAP-RGL2 expressed in TAP-RGA, TAP-RGL1, or TAP-RGL2 transgenic *Arabidopsis* plants. Bound proteins were washed, separated on SDS-PAGE, and immunoblotted with the anti-c-myc antibody (α-myc). The input lane shows the expression level of TAP-RGA, TAP-RGL1, or TAP-RGL2 expressed in TAP-RGA, TAP-RGL1, or TAP-RGL2 transgenic plants. The positions of purified MBP, MBP-MYB21 and MBP-MYB24 on SDS-PAGE are indicated with asterisks (stained by Coomassie blue). The original data can be viewed from Additional file 4: Figure S3a-d

We fused MYB21 with LexA DNA binding domain (BD), and found that BD-MYB21 showed strong auto-activation in yeast (Additional file 2: Figure S1a). We further truncated MYB21 into MYB21NT containing R2R3 DNA binding domain and MYB21CT including NYW^G^/_S_^M^/_V_DD^I^/_L_W^S^/_P_ motif (Fig. 1a), and found that MYB21NT lost strong auto-activation (Additional file 2: Figure S1a). MYB21NT was used as bait to screen MYB21 interaction proteins in *Arabidopsis* cDNA library in Y2H system. The DELLA protein RGA is one of the putative interaction clones.

We further detected the interactions of MYB21NT with the five *Arabidopsis* DELLAs in Y2H system, and found that BD-fused MYB21NT interacted with activation domain (AD)-fused RGA, GAI, RGL1, RGL2, and RGL3 (Fig. 1b), whereas no interaction was detected in negative controls (Fig. 1b and Additional file 3: Figure S2a). MYB21 and MYB24 are homologs with 67.7% identity at amino acid level [52]. We next investigated whether MYB24 can also interact with DELLAs. BD-fused MYB24 showed strong auto-activation in yeast (Additional file 2: Figure S1b). We truncated MYB24 into MYB24NT (Fig. 1a), which did not show auto-activation (Additional file 2: Figure S1b). As shown in Fig. 1b, MYB24NT also interacted with RGA, GAI, RGL1, RGL2, and RGL3 in yeast.

Full-length of DELLAs with BD domain showed strong auto-activation in yeast, therefore, we truncated DELLAs into L and R domains (RGA-L and RGL2-L; RGA-R, GAI-R, RGL1-R, RGL2-R, and RGL3-R) (Fig. 1c), and R domains lost strong auto-activation. MYB21 and MYB24 were respectively fused with AD domain, and showed no auto-activation in yeast (Additional file 3: Figure S2b). The Y2H results in Fig. 1d showed that RGA-R, GAI-R, RGL1-R, RGL2-R, and RGL3-R interact with MYB21 and MYB24 respectively in yeast.

We next performed pull-down assay to verify the interactions of MYB21 and MYB24 with DELLAs in vitro. Maltose binding protein (MBP), MBP-fused MYB21 (MBP-MYB21) and MYB24 (MBP-MYB24) were expressed in *Escherichia coli* and purified by amylose resin. DELLA proteins RGA, RGL1 and RGL2 were extracted from transgenic *Arabidopsis* expressing tandem affinity purification (TAP) tag-fused DELLA proteins (TAP-RGA, TAP-RGL1 and TAP-RGL2) [41]. MBP, MBP-MYB21 and MBP-MYB24 were incubated with TAP-RGA extracts expressing TAP-RGA, and then separated on SDS-PAGE for immunoblotting with anti-c-myc antibody. The result showed that MBP-MYB21 and MBP-MYB24 could efficiently pull down TAP-RGA, but the negative control MBP could not (Fig. 1e), indicating that RGA interacts with MYB21 and MYB24. In addition, we also observed that DELLA proteins RGL1 and RGL2 interact with MYB21 and MYB24 in pull -down assay (Fig. 1f and g).

Taken together, the Y2H assay, and pull-down assay consistently demonstrate that DELLAs interact with the R2R3 MYB transcription factors (MYB21 and MYB24), implying that these two transcription factors function as direct targets of DELLA proteins.

### R2R3 domains of MYB21 and MYB24 are involved in interactions with DELLAs

We further investigated interactions of DELLA R fragments with MYB21NT, MYB24NT, MYB21CT, and MYB24CT. As shown in Fig. 1d, RGA-R, GAI-R, RGL1-R, RGL2-R, and RGL3-R interacted with MYB21NT and MYB24NT, but not with MYB21CT and MYB24CT, whereas no interaction was detected in negative controls (Fig. 1d and Additional file 3: Figure S2b), indicating that DELLAs interact with R2R3 DNA binding domains of MYB21 and MYB24.

We also examined whether L or R domains of DELLA proteins interact with MYB21NT and MYB24NT. Y2H results suggested that MYB21NT and MYB24NT interact with both L parts and R parts of RGA and RGL2 (Fig. 1b), while no interaction was detected in negative controls (Fig. 1b and Additional file 3: Figure S2a), indicating that both N-terminus and C-terminus of DELLAs interact with MYB21 and MYB24.

### DELLA and JAZ proteins synergistically inhibit transcriptional function of MYB21 and MYB24

Having shown that DELLAs interact with MYB21 and MYB24, we then performed an *Arabidopsis* protoplast transient expression assay [53] using the GAL4 DNA binding domain (GAL4DB) and its binding sites [GAL4(4X)-D1–3(4X)] to test whether DELLAs could influence the transcriptional function of MYB21 and MYB24.

**Fig. 2:**
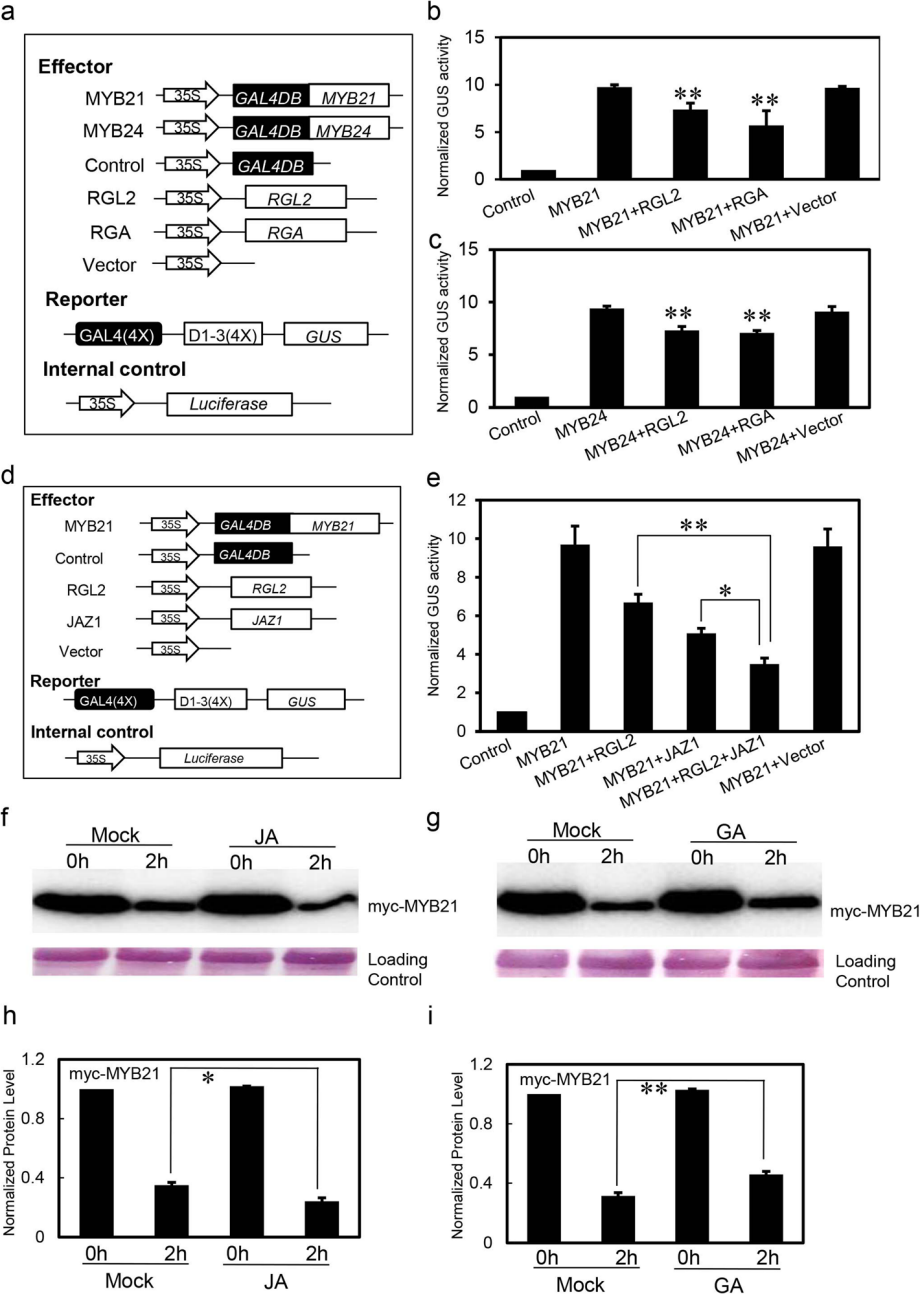
DELLAs and JAZs coordinately inhibit the transcriptional function of MYB21. **a** The schematic diagram shows the constructs used in the transient expression assays of **b** and **c**. **b** and **c** Transient expression assays show that RGA and RGL2 inhibit transcriptional function of MYB21 **b** and MYB24 **c**. The GUS reporter and the internal control luciferase (LUC) were cotransformed with the indicated constructs. Data are means (±SE) of three biological replicates. Asterisks represent Student’s t-test significance compared with the MYB21 (**, *P* < 0.01). **d** Schematic diagram of the constructs used in transient expression assays in **e**. **e** Transient expression assays show that both RGL2 and JAZ1 proteins could significantly repress transcriptional function of MYB21. Data are means (±SE) of three biological replicates. Asterisks represent Student’s t-test significance between pairs indicated with brackets (*, *P* < 0.05; **, *P* < 0.01). **f** and **g** Effects of jasmonate and gibberellin on the protein stability of MYB21. MYB21 (myc-MYB21) transiently expressed in *N. benthamiana* was extracted from tobacco leaves, and then incubated without (Mock) or with methyl jasmonate (JA) **f**, gibberellin (GA) **g**, for the indicated time (hours). Then myc-MYB21 was detected by immunoblot using anti-c-myc antibody. The PVDF membrane was stained with Memstain to serve as loading control. The original data can be viewed from Additional file 4: Figure S3e-f. **h** and **i** Quantitative analysis of myc-MYB21 protein level in **f** and **g**. Data are means (±SE) of three biological replicates. Asterisks represent Student’s t-test significance between pairs indicated with brackets (*, *P* < 0.05; **, *P* < 0.01). The abundance of myc-MYB21 with mock treatment for 0 h was set to 1.0

MYB21 and MYB24 were respectively fused with GAL4DB vector and served as effectors. The reporter was the *GUS* (β-glucuronidase) gene controlled by four copies of GAL4 DNA binding site [GAL4(4x)-D1–3(4x)], and the internal control was 35S promoter-driven *firefly luciferase* (LUC) gene (Fig. 2a). The DELLA genes *RGA* and *RGL2* were respectively cloned into pGreen62 vector (Fig. 2a). As shown in Fig. 2b, expression of GAL4DB-MYB21 together with the GUS reporter could enhance the GUS/LUC ratio, while coexpression of RGA or RGL2 with GAL4DB-MYB21 repressed the transcriptional function of MYB21. We also observed that RGA and RGL2 inhibit the transcriptional function of MYB24 (Fig. 2c).

As both JAZs and DELLAs interact with MYB21 and MYB24 (Fig. 1) [32], and DELLAs could repress these two factors transcriptional function (Fig. 2b and c), we further investigated whether JAZs or DELLAs and JAZs synergistically regulate the transcriptional function of MYB21 and MYB24. We used MYB21 as the representative (Fig. 2d) and found that RGL2 or JAZ1 alone repressed the transcriptional activity of MYB21 (Fig. 2e). We further discovered that coexpression of RGL2 and JAZ1 exhibited a much stronger inhibition of the transcriptional activity of MYB21 compared with RGL2 or JAZ1 alone (Fig. 2e). These results revealed that DELLAs and JAZs coordinately repress the transcription activity of MYB21.

We also explored a cell-free assay system [54] to discuss whether the MYB21 protein level is regulated by GA or JA through analysis of *N. benthamiana* leaves transiently expressed myc-taged MYB21 (myc-MYB21). As the results shown in Fig. 2f and h, the myc-MYB21 protein level was decreased to ~ 33% after 2 h treatment with mock, while JA treatment promoted the degradation of myc-MYB21 protein (~ 24% of the myc-MYB21 level without treatment). However, GA could delay the degradation of myc-fused MYB21 (~ 46% of the myc-MYB21 level without treatment) (Fig. 2g and i). These results indicated that GA and JA may play an opposite role in regulating the stability of MYB21 protein.

### DELLAs and JAZs converge on MYB21 and MYB24 to regulate filament elongation

As both JAZs and DELLAs target MYB21 and MYB24, we further explore the cross-talk of JA and GA in the regulation of filament elongation.

**Fig. 3:**
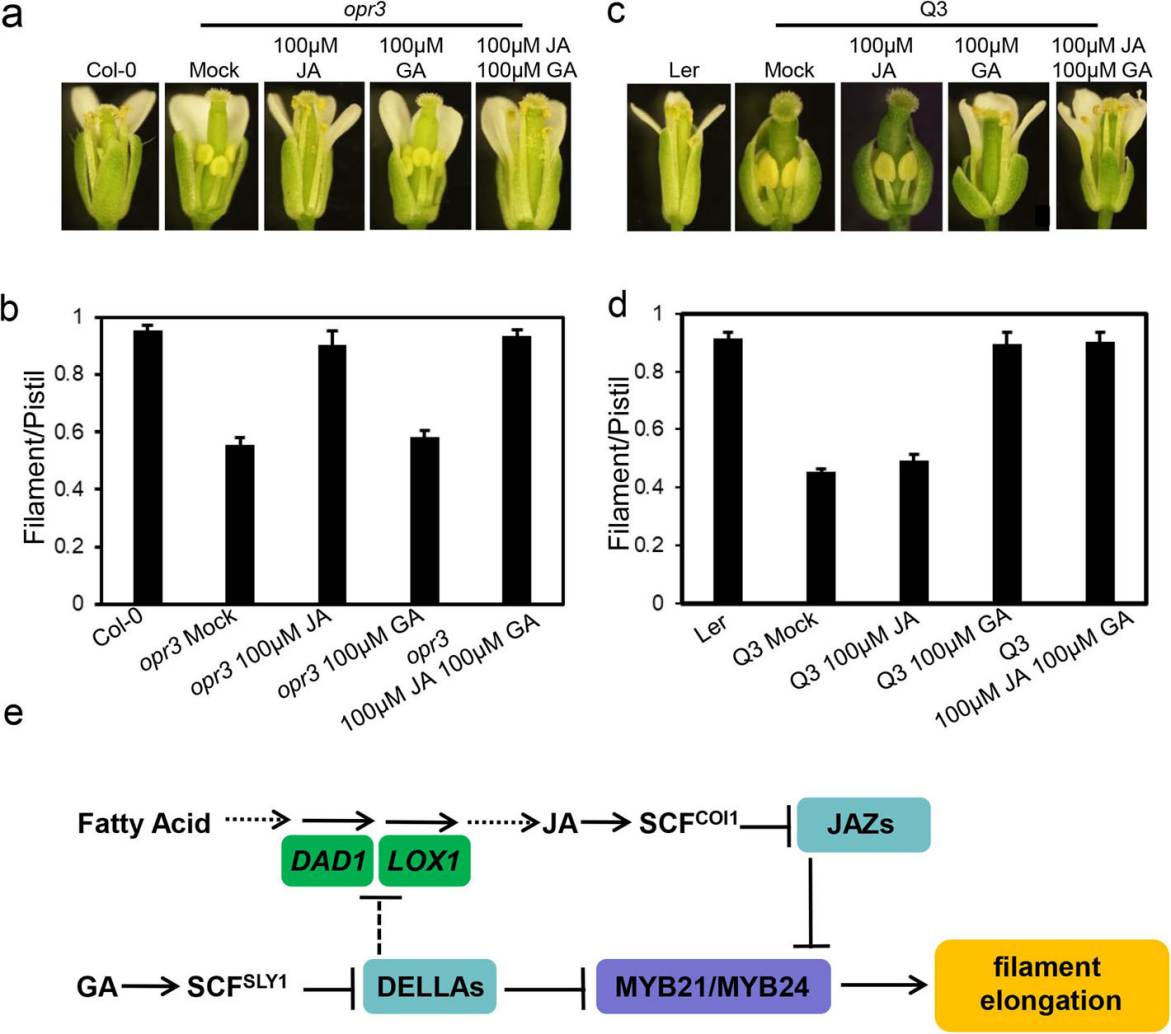
DELLAs and JAZs converge on MYB21 and MYB24 to regulate filament elongation. **a** Comparison of flowers at floral stage 13 in Col-0 and *opr3* treated without (Mock) or with methyl jasmonate (JA), gibberellin (GA), or JA plus GA for the indicated concentration. **b** The ratio of filament length and pistil length at floral stage 13 in the indicated genotypes. Data are means (±SE) of three biological replicates. **c** Comparison of flowers at floral stage 13 in Landsberg *erecta* (Ler) wild-type and *ga1–3 gai-t6 rga-t2 rgl1–1* (Q3) treated without (Mock) or with methyl jasmonate (JA), gibberellin (GA), or JA plus GA for the indicated concentration. **d** The ratio of filament length and pistil length at floral stage 13 in the indicated genotypes. Data are means (±SE) of three biological replicates. **e** A simplified model for the crosstalk between jasmonate and gibberellin in regulating filament elongation. JAZs interact with and inhibit the transcriptional function of MYB21 and MYB24 to suppress filament elongation (Song et al. 2011; Qi et al. 2015). DELLAs inhibit the expression of JA-biosynthesis gene *DAD1* and *LOX1* (Cheng et al. 2009), and as well as interact with and attenuate MYB21 and MYB24 to inactivate downstream genes and repress filament elongation. JA and GA signal respectively induce degradation of JAZs and DELLAs to derepress MYB21 and MYB24, and synergistically modulate filament elongation in *Arabidopsis*

Filaments in JA-deficient mutant *opr3* at floral stage 13 are much shorter than that in wild type, indicating that stabilized JAZ proteins attenuate the function of MYB21 and MYB24 to repress filament elongation, and JA treatment could restore the filament elongation of *opr3* (Fig. 3a and b). Previous studies showed that GA could induce degradation of DELLA proteins [46, 55]. We tested whether GA treatment could release DELLA-targeted MYB21 and MYB24 to enhance filament growth in *opr3*. We further measured the ratio of filament to pistil length of *opr3* treated with GA. As shown in Fig. 3a and b, GA treatment could only very slightly rescue the filament/pistil ratio of *opr3*, indicating that majority of MYB21 and MYB24 are inhibited by JAZs in *opr3*, and that the released MYB21 and MYB24 by GA treatment were not enough to rescue the filament elongation of *opr3*, whereas treatment with both JA and GA recovered filament elongation of *opr3* (Fig. 3a and b).

RGL2 protein alone could repress the function of MYB21 and MYB24 to control filament elongation in Q3 (*ga1–3 gai-t6 rgl1–1 rga-t2*, wild type for RGL2 and RGL3) [52]. Application of GA could induce RGL2 degradation and restored the filament elongation of Q3 (Fig. 3c and d). We further explored whether degradation of JAZs by JA treatment could release enough MYB21 and MYB24 to rescue the filament growth of Q3. As shown in Fig. 3c and d, JA treatment could very weakly recover the ratio of filament and pistil length of Q3, indicating that RGL2 alone in Q3 is able to dominantly inhibit the function of MYB21 and MYB24 to repress the filament elongation. Exogenous application of both GA and JA could also restore the filament elongation of Q3 (Fig. 3c and d).

## Discussion

Both GA and JA act antagonistically to regulate hypocotyl elongation and root growth [56, 57], and synergistically to mediate stamen development, trichome development and sesquiterpene production [43, 52, 58]. In this work, we further present a new model that provides a synergistic mechanism for GA and JA signaling in filament elongation. In this model, both DELLAs and JAZs interact with R2R3 MYB transcription factors MYB21 and MYB24 to repress their transcription activity, while GA and JA induce the degradation of DELLAs and JAZs, respectively, to release and coordinately activate MYB21 and MYB24, and synergistically regulate filament elongation (Fig. 3e).

Consistent with this synergistic regulatory mechanism, accumulation of JAZs in the JA biosynthesis mutant (*opr3*), and the DELLA protein RGL2 in GA-deficient mutant Q3, respectively interact with MYB21 and MYB24, and repress filament elongation. Exogenous GA treatment of *opr3* or JA treatment of Q3 could not significantly recover the filament elongation (Fig. 3a-d), suggesting that JAZs in *opr3* and RGL2 in Q3 are efficient to inhibit the function of MYB21 and MYB24 to control filament elongation.

A recent study revealed that IIIe bHLH transcription factors (MYC2, MYC3, MYC4 and MYC5) act as JAZs-targeted proteins to redundantly regulate filament elongation, and that these four factors interact with MYB21 and MYB24 to form a MYB-MYC complex to regulate filament elongation [38]. Having shown that DELLAs interact with and repress the transcription activity of the MYB components of the MYB-MYC complex (Figs. 1 and 2), it will be interesting to investigate whether DELLAs are able to interact with and attenuate the transcriptional function of the bHLH components (MYC2, MYC3, MYC4 and MYC5) of the MYB-MYC complex to regulate filament elongation in *Arabidopsis*.

Previous studies revealed that JA induces the expression of *MYB21* and *MYB24* [33, 34], and that GA promotes the JA biosynthesis to activate the mRNA level of *MYB21* and *MYB24* [52]. We also explored whether JA and GA affect the protein stability of MYB21. As shown in Fig. 2f-i, JA and GA respectively could promote, or delay the degradation of MYB21. It indicated that the post-transcription regulation mechanism of MYB21 is sophisticated, which would contribute to the delicate dynamic stamen development and need further investigation.

## Conclusions

In conclusion, we found that MYB21 and MYB24 are the direct targets of DELLAs, and that DELLA and JAZ proteins synergistically repress the transcription activity of MYB21 and MYB24 to repress filament elongation.

## Methods

### Plant materials and growth conditions

The *Arabidopsis thaliana* mutants *opr3* and Q3 were previously described [29, 52], and respectively donated by professor John Browse (Washington State University) and professor Jinrong Peng (Zhejiang University). The *Arabidopsis thaliana* transgenic plants TAP-RGA, TAP-RGL2 and TAP-RGL1 were previously described [41], and donated by professor Xingwang Deng (Peking University). *Arabidopsis* seeds were surface sterilized with 20% bleach for 10 min, plated on Murashige and Skoog (MS) medium (Sigma-Aldrich) supplied with 3% sucrose, kept at 4 °C for 3 days in the dark, and then plated to growth house with 16 h-light (22 °C to 24 °C)/8 h-dark (16 °C to 19 °C) photoperiod. Seeds of Q3 were immerged in 100 μM GA_3_ at 4 °C for 7 days before sowing. *Nicotiana benthamiana* seeds were donated by professor Yule Liu (Tsinghua University), and cultivated at 22 °C–28 °C under 16 h-light/8 h-dark photoperiod.

### Y2H screening and Y2H assay

The Y2H screening method and Y2H assay was previously described [32]. For Y2H assay, MYB21, MYB24, RGA, GAI, RGL1, RGL2, RGL3, and their related domains were inserted into pLexA or pB42AD vectors respectively. The indicated construct pairs were co-transformed into yeast strains EGY48, and plated on SD agar medium with -His/−Trp/−Ura DO supplement (Clontech) at 30 °C for 4–5 days. The yeast transformants was resuspended with SD/−His-Trp-Ura liquid medium and cultured at 30 °C for 24 h, subsequently harvested and resuspended with distilled water. 5 μl of the indicated suspension was dropped into 96-well plates containing 2% Gal/1% raffinose/SD/−Ura/−His/−Trp/−Leu/X-β-Gal medium. Y2H images were taken 2 days after incubation at 30 °C. The primers for the vector construction are listed in Additional file 1: Table S1.

### Pull-down assay

The coding region of *MYB21*, and *MYB24* were cloned into the pMAL-c5x (NEB) vector to generate MBP fused MYB21 and MYB24 respectively. The MBP, MBP-fused MYB21 and MYB24 were expressed in *E. coli* and purified by amylose resin beads.

The method for pull-down assay was previously described [7]. For pull-down assay, 5 g of 14-d-old TAP-RGA, TAP-RGL2, and TAP-RGL1 transgenic seedlings respectively collected, and total protein was extracted using RB buffer (25 mM imidazole, 100 mM NaCl, 50 mM Tris-Cl, pH 7.8, EDTA-free complete miniprotease inhibitor cocktail, 0.1% [v/v] Tween20, 10% [v/v] glycerol, and 20 mM 2-mercaptoethanol), and was concentrated to 400 μL. 50 mg of purified MBP, MBP-MYB21 and MBP-MYB24 was respectively incubated with 200 μL amylose resin beads with gentle rotation at 4 °C for 2 h, and washed five times using RB buffer. Subsequently added 100 μL concentrated total proteins containing TAP-RGA, TAP-RGL2, or TAP-RGL1 for 2 h at 4 °C. After washing five times with RB buffer, the mixture was denatured in SDS loading buffer. The samples were separated on SDS-PAGE, and immunoblotted using anti-c-myc antibody (Abmart).

### Protoplast transfection assay

For transient expression assay, the *Arabidopsis MYB21* and *MYB24* were amplified and fused with the GAL4DB vector through *Sma*I and *Sal*I sites, and *RGA*, *RGL2* and *JAZ1* were respectively cloned into the pGreenII 62-SK [59]. The *GUS*, and *the firefly LUC* gene was respectively controlled by four copies of upstream GAL4DB binding sites (GAL4(4x)-D1–3(4x)) and 35S promoter, and acted as a reporter and the internal control respectively [53]. *Arabidopsis* mesophyll protoplasts preparation and transfection were described as previously [60]. Primers used for plasmid construction are listed in Additional file 1: Table S1.

### Protein degradation assay

*N. benthamiana* leaves were infiltrated with *Agrobacterium* strains harboring myc-MYB21. 50 h after infiltration, 3 g of *Agrobacterium*-infiltrated leaves was collected. The total protein was extracted using RB buffer and incubated at 22 °C for indicated time periods without or with 100 μM methyl-jasmonate (MeJA), 100 μM GA_3_, then separated by SDS-PAGE, transferred to PVDF membrane, and detected with anti-c-myc antibody (Abmart). The protein level of MYB21 was quantified by the software from FluorChem M MultiFluor System (Alpha). The experiment was repeated for three independent biological replicates.

### Measurement of pistil and filament length

For this experiment, the pistil and one of the four longer filaments of 10 flowers at floral stage 13 [61] of each genotype were collected and measured under the microscope. Young flowers buds of *opr3* and Q3 were treated with mock, 100 μM methyl jasmonate, 100 μM GA_3_, or 100 μM methyl jasmonate plus 100 μM GA_3_ twice a day for detecting the ratio of filament and pistil length. The experiment was repeated for three independent biological replicates.

### Accession numbers

The Arabidopsis Genome Initiative numbers for the genes mentioned in this article are as follows: GAI (AT4G02780), RGA (AT2G01570), RGL1 (AT1G66350), RGL2 (AT3G03450), RGL3 (AT5G17490), JAZ1 (AT1G19180), MYB21 (AT3G27810), and MYB24 (AT5G40350).

## Supplementary information

**Additional file 1:** **Table S1.** Primers Used for Vector Construction

**Additional file 2:** **Figure S1.** Detection of Auto-activation for MYB21, MYB21NT, MYB24, and MYB24NT in Y2H Experiments. MYB21, MYB21NT, MYB24, and MYB24NT were individually fused with the LexA DNA binding domain (BD) in pLexA. The full-length of MYB21 and MYB24 with BD domain exhibited auto-activation, while N-terminal of MYB21 (MYB21NT) and MYB24 (MYB24NT) with BD domain lost auto-activation. Auto-activation (represented by blue color) were assessed on 2% Gal/1% raffinose/SD/−Ura/−His/−Trp/−Leu/X-β-Gal medium.

**Additional file 3:** **Figure S2.** Negative Controls for the Y2H Experiments. No interaction was detected after co-expression of pB42AD-RGA/RGA-L/RGA-R/GAI/RGL1/RGL2/RGL2-L/RGL2-R/RGL3/MYB21/MYB21NT/MYB21CT/MYB24/MYB24NT/MYB24CT with the BD domain in pLexA empty vector. Interactions were assessed on 2% Gal/1% raffinose/SD/−Ura/−His/−Trp/−Leu/X-β-Gal medium.

**Additional file 4:** **Figure S3.** Source data for Figs. 1e-g and 2f-g. (a-c) Red frame in Figures a-c respectively displayed the source data for Figs. 1e-g. (d) Full scan of SDS-PAGE gel shown in Figs. 1e-g. Red frame from left to right displayed the source data for Fig. 1e, g, and f, respectively. Asterisks indicated the positions of purified MBP, MBP-MYB21 and MBP-MYB24. (e) Full scan of the results shown in Figs. 2f-g. Red frame from left to right respectively displayed the source data for Fig. 2f, and g. (f) Red frame from left to right respectively displayed the source data for Figs. 2f, and 2 g.

## Data Availability

All the data supporting the conclusions of this article are included in figures and additional files. The data and plant materials are available from the corresponding author on reasonable request.

## Abbreviations

COI1: Coronatine Insensitive 1
GA: Gibberellin
GAI: Gibberellic Acid Insensitive
JA: Jasmonate
JAZ: JASMONATE ZIM-domain
MBP: Maltose binding protein
RGA: Repressor Of GA1–3
RGL1: RGA-like1
TAP: Tandem affinity purification
