# Supplementary material for: The DELLA proteins interact with MYB21 and MYB24 to regulate filament elongation in Arabidopsis
Source: BMC Plant Biol. 2020 Feb 7;20:64. doi: 10.1186/s12870-020-2274-0 (PMC7006197; doi:10.1186/s12870-020-2274-0)
Supplement: Supplementary file 1 — Additional file 1: Table S1. Primers Used for Vector Construction [file 12870_2020_2274_MOESM1_ESM.doc]

Supplemental Table 1. Primers Used for Vector Construction

| MYB21 AD | Forward | cgccaattgatggagaaaagaggaggaggaag |
| --- | --- | --- |
| MYB21 AD | Reverse | cccgctcgagtcaattaccattcaataaatg |
| MYB21NT AD/BD | Forward | cgccaattgatggagaaaagaggaggaggaag |
| MYB21NT AD/BD | Reverse | cccgctcgagtcacgaatagttaccatagttg |
| MYB21CT AD | Forward | cgccaattgacaacaacatcgtccgttggatc |
| MYB21CT AD | Reverse | cccgctcgagtcaattaccattcaataaatg |
| MYB24 AD | Forward | ggggaattcatggagaaaagagaaagtagtg |
| MYB24 AD | Reverse | cccgctcgagttaattaccattatatatattc |
| MYB24NT AD/BD | Forward | ggggaattcatggagaaaagagaaagtagtg |
| MYB24NT AD/BD | Reverse | ccgctcgagtcaataattaccataattaagc |
| MYB24CT AD | Forward | ggggaattcacgacgaccgttggatcacaaa |
| MYB24CT AD | Reverse | cccgctcgagttaattaccattatatatattc |
| RGA-R BD/AD | Forward | ccccaattggcgggtgagtcaactcgttctg |
| RGA-R BD/AD | Reverse | ccgctcgagtcagtacgccgccgtcgagagttt |
| GAI-R BD | Forward | ggggaattcacggctgagtcaactcggcatgttgtc |
| GAI-R BD | Reverse | acgcgtcgacctaattggtggagagtttccaagc |
| RGL1-R BD | Forward | ccccaattgtctacgcgctctgtggtggttttgg |
| RGL1-R BD | Reverse | acgcgtcgacttattccacacgattgattcgcca |
| RGL2-R BD/AD | Forward | ccccaattgtcggacgagtcaactcggtccgtgg |
| RGL2-R BD/AD | Reverse | agcgtcgactcaggcgagtttccacgccgaggttg |
| RGL3-R BD | Forward | ccccaattggagtcaactcgttccgtggtgctt |
| RGL3-R BD | Reverse | acgcgtcgacctaccgccgcaactccgccgctagt |
| RGA AD | Forward | ccccaattgatgaagagagatcatcaccaattccaaggt |
| RGA AD | Reverse | acgcgtcgactcagtacgccgccgtcgagagtttccaagc |
| RGA-L AD | Forward | ccggaattcatgaagagagatcatcaccaattc |
| RGA-L AD | Reverse | acgcgtcgactcaaccaacttcatgaagatgatc |
| GAI AD | Forward | ccggaattcatgaagagagatcatcatcatcatc |
| GAI AD | Reverse | acgcgtcgacctaattggtggagagtttccaagc |
| RGL1 AD | Forward | ccccaattgatgaagagagagcacaaccaccgt |
| RGL1 AD | Reverse | acgcgtcgacttattccacacgattgattcgcca |
| RGL2-L AD | Forward | cgccaattgatgaagagaggatacggagaaacatggg |
| RGL2-L AD | Reverse | agagtcgacttactgttgaagcgaatctgaattctccg |
| RGL2 AD | Forward | cgccaattgatgaagagaggatacggagaaacatggg |
| RGL2 AD | Reverse | agcgtcgactcaggcgagtttccacgccgaggttg |
| RGL3 AD | Forward | ccccaattgatgaaacgaagccatcaagaaacg |
| RGL3 AD | Reverse | acgcgtcgacctaccgccgcaactccgccgctagt |
| MBP-MYB21 | Forward | aaaacatatgatggagaaaagaggaggaggaag |
| MBP-MYB21 | Reverse | aaagcggccgcttagtggtggtggtggtggtgattaccattcaataaatgcattg |
| MBP-MYB24 | Forward | aaaacatatgatggagaaaagagaaagtagtg |
| MBP-MYB24 | Reverse | aaagcggccgcttagtggtggtggtggtggtgattaccattatatatattcatggg |
| MYB21-GAL4DB | Forward | agacccggggatggagaaaagaggaggagg |
| MYB21-GAL4DB | Reverse | acgcgtcgactcaattaccattcaataaatgc |
| MYB24-GAL4DB | Forward | agacccggggatggagaaaagagaaagtagtg |
| MYB24-GAL4DB | Reverse | acgcgtcgacttaattaccattatatatattcatg |
| RGL2-pGreenII 62-SK | Forward | agacccgggatgaagagaggatacggag |
| RGL2-pGreenII 62-SK | Reverse | agagtcgactcaggcgagtttccacgccgag |
| RGA-pGreenII 62-SK | Forward | atcgagctcatgaagagagatcatcacca |
| RGA-pGreenII 62-SK | Reverse | agagtcgactcagtacgccgccgtcgagag |
| JAZ1-pGreenII 62-SK | Forward | atcgagctcatgtcgagttctatggaatg |
| JAZ1-pGreenII 62-SK | Reverse | agagtcgactcatatttcagctgctaaac |
| myc MYB21 | Forward | agacccgggatggagaaaagaggaggaggaag |
| myc MYB21 | Reverse | atcgagctctcaattaccattcaataaatgca |
