# Supplementary figures and images for: The DELLA proteins interact with MYB21 and MYB24 to regulate filament elongation in Arabidopsis
Source: BMC Plant Biol. 2020 Feb 7;20:64. doi: 10.1186/s12870-020-2274-0 (PMC7006197; doi:10.1186/s12870-020-2274-0)

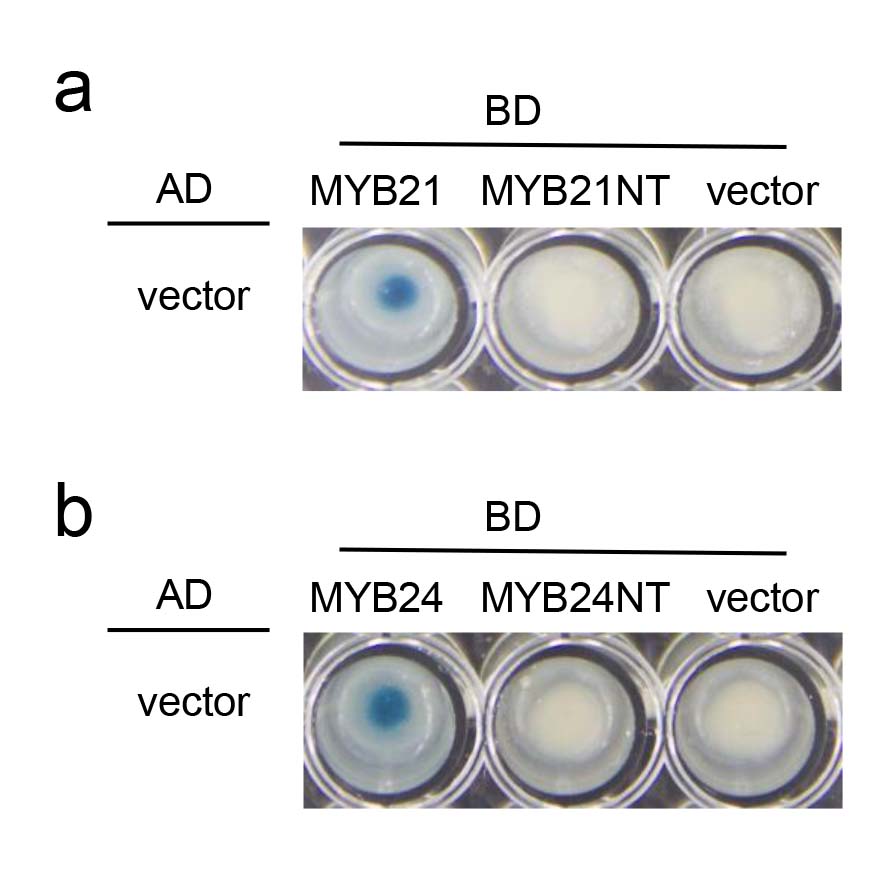

Supplement: Supplementary file 2 — Additional file 2: Figure S1. Detection of Auto-activation for MYB21, MYB21NT, MYB24, and MYB24NT in Y2H Experiments. MYB21, MYB21NT, MYB24, and MYB24NT were individually fused with the LexA DNA binding domain (BD) in pLexA. The full-length of MYB21 and MYB24 with BD domain exhibited auto-activation, while N-terminal of MYB21 (MYB21NT) and MYB24 (MYB24NT) with BD domain lost auto-activation. Auto-activation (represented by blue color) were assessed on 2% Gal/1% raffinose/SD/−Ura/−His/−Trp/−Leu/X-β-Gal medium. [file 12870_2020_2274_MOESM2_ESM.jpg]

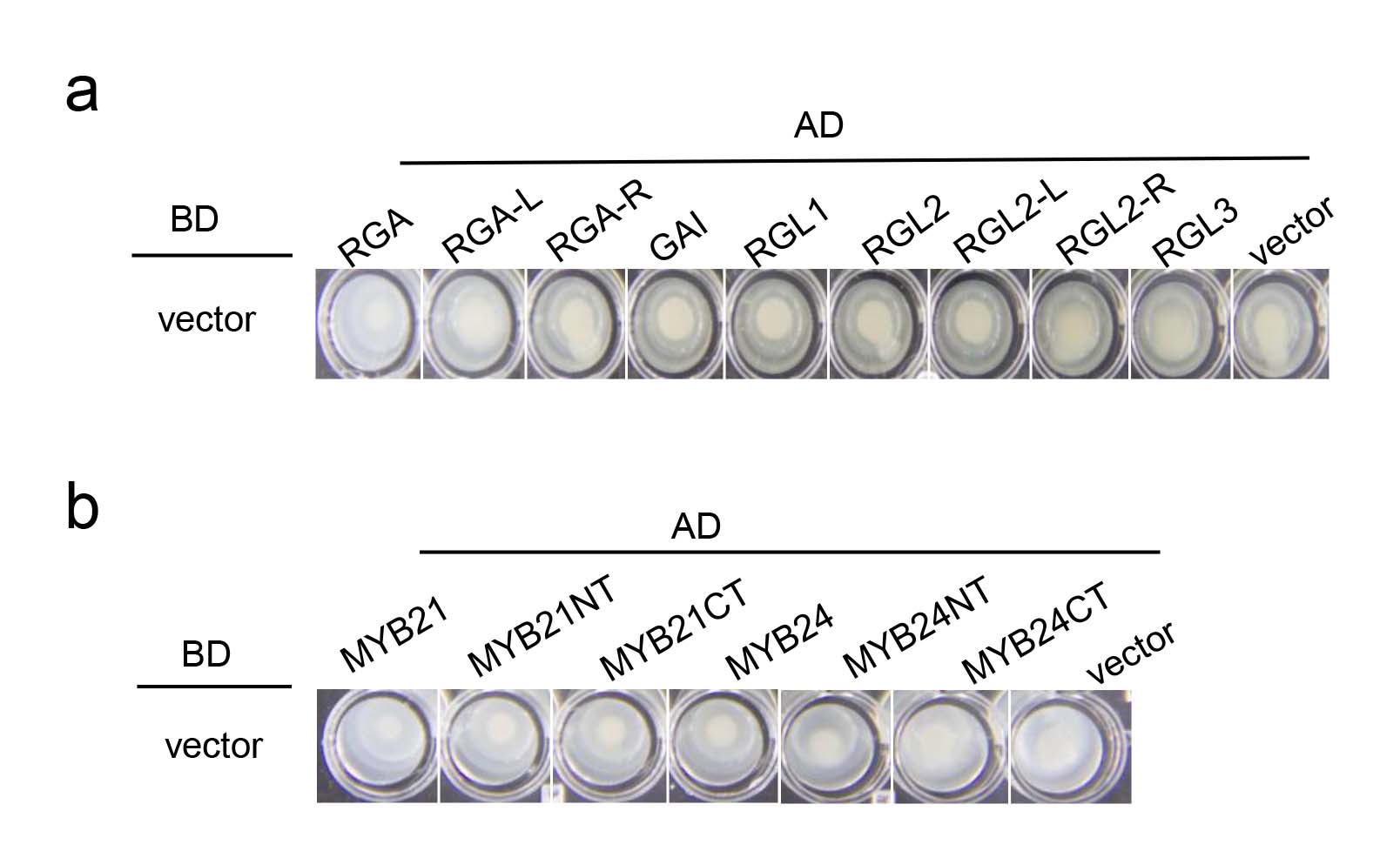

Supplement: Supplementary file 3 — Additional file 3: Figure S2. Negative Controls for the Y2H Experiments. No interaction was detected after co-expression of pB42AD-RGA/RGA-L/RGA-R/GAI/RGL1/RGL2/RGL2-L/RGL2-R/RGL3/MYB21/MYB21NT/MYB21CT/MYB24/MYB24NT/MYB24CT with the BD domain in pLexA empty vector. Interactions were assessed on 2% Gal/1% raffinose/SD/−Ura/−His/−Trp/−Leu/X-β-Gal medium. [file 12870_2020_2274_MOESM3_ESM.jpg]

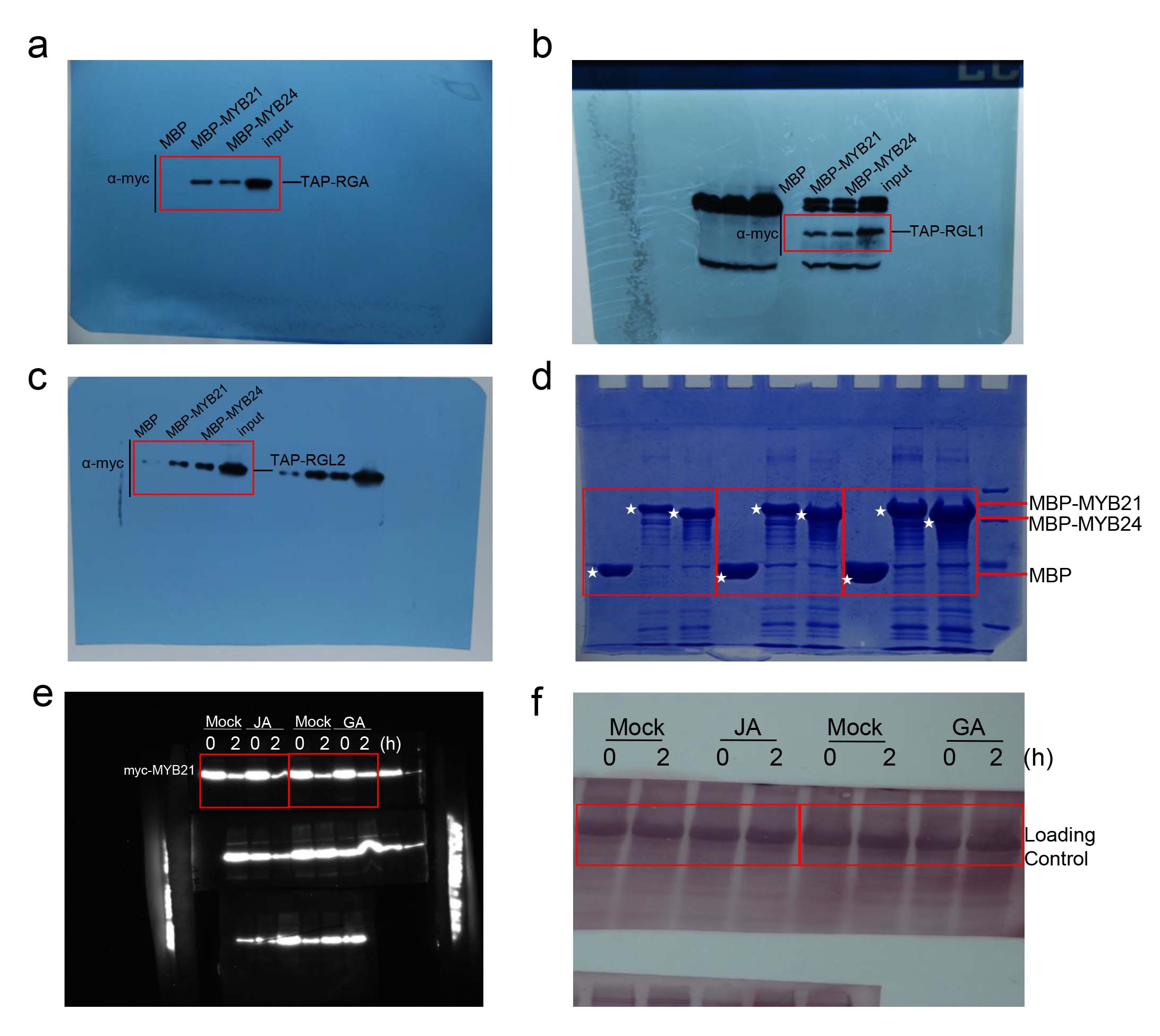

Supplement: Supplementary file 4 — Additional file 4: Figure S3. Source data for Figs. 1e-g and 2f-g. (a-c) Red frame in Figures a-c respectively displayed the source data for Figs. 1e-g. (d) Full scan of SDS-PAGE gel shown in Figs. 1e-g. Red frame from left to right displayed the source data for Fig. 1e, g, and f, respectively. Asterisks indicated the positions of purified MBP, MBP-MYB21 and MBP-MYB24. (e) Full scan of the results shown in Figs. 2f-g. Red frame from left to right respectively displayed the source data for Fig. 2f, and g. (f) Red frame from left to right respectively displayed the source data for Figs. 2f, and 2 g. [file 12870_2020_2274_MOESM4_ESM.jpg]
